# Ethanol Alleviates Amyloid-β-Induced Toxicity in an Alzheimer’s Disease Model of *Caenorhabiditis elegans*

**DOI:** 10.3389/fnagi.2021.762659

**Published:** 2021-11-12

**Authors:** Shuju Bai, Wenbo Wang, Zhiwei Zhang, Mengyao Li, Zehan Chen, Jiuqiao Wang, Yanlin Zhao, Lu An, Yuxiang Wang, Shu Xing, Xueqi Fu, Junfeng Ma

**Affiliations:** ^1^National Engineering Laboratory for AIDS Vaccine, School of Life Sciences, Jilin University, Changchun, China; ^2^School of Mathematics, Jilin University, Changchun, China; ^3^Key Laboratory for Molecular Enzymology and Engineering, The Ministry of Education, School of Life Sciences, Jilin University, Changchun, China

**Keywords:** Alzheimer’s disease, amyloid-β, *Caenorhabiditis elegans*, ethanol, *DAF-16*, lysosome

## Abstract

Amyloid-β, a hallmark of Alzheimer’s disease, forms toxic intracellular oligomers and extracellular senile plaques resulting in neuronal toxicity. Ethanol is widely consumed worldwide. Moderate ethanol consumption has numerous benefits in humans. We found that ethanol could significantly extend the lifespan of *Caenorhabiditis elegans* in a previous study. Based on that study, we tested the effect of ethanol on Alzheimer’s disease transgenic *Caenorhabiditis elegans* strain CL4176, which expresses amyloid-β1-42 peptide in body wall muscle cells. Ethanol delayed paralysis and reduced amyloid-β oligomers in *Caenorhabiditis elegans* worms of the CL4176 strain. Moreover, ethanol could induce the nuclear translocation of DAF-16 in the nematodes. However, in worms that were fed *daf-16* RNAi bacteria, ethanol no longer delayed the paralysis. The qPCR assays showed that ethanol increases the expression of *daf-16*, *hsf-1* and their common target genes- small heat shock protein genes. In addition, we also found that ethanol could increase lysosome mass in the CL4176 worms. In summary, our study indicated that ethanol attenuated amyloid-β toxicity in the Alzheimer’s disease model of *Caenorhabiditis elegans* via increasing the level of lysosomes to promote amyloid-β degradation and upregulating the levels of small heat shock protein genes to reduce amyloid-β aggregation.

## Introduction

Alzheimer’s disease (AD), a progressive neurodegenerative disorder, is the main cause of dementia and is one of the major health care challenges of the twenty-first century (Scheltens et al., 2016). Neurofibrillary tangles and senile plaques, consisting of hyperphosphorylated tau protein and insoluble amyloid-β (Aβ) peptide, respectively, are the main pathological features in the brains of patients with AD (Qiu et al., 2015). According to the amyloid cascade hypothesis, amyloid precursor protein is cleaved by β-secretase and γ-secretase to form Aβ, which easily aggregates into large oligomers and is responsible for both sporadic and familial AD (Haass and Selkoe, 2007; Lublin and Link, 2013). Many studies suggest that Aβ1-42 is more prone to accumulation, more neurotoxic, and more directly related to AD pathology than Aβ1-40. Therefore, many researchers have focused on Aβ1-42 as a biomarker to study AD progression and therapy. *Caenorhabiditis elegans* is a transparent, 1mm long roundworm. Because of their short lifespan and simple physical structure, nematodes are extremely well-suited to study aging diseases in humans (Shen et al., 2018), and many researchers have used a *Caenohabiditis elegans* model expressing Aβ to study AD. The transgenic nematode strain CL4176 was built to express human Aβ1-42 peptide under the special *myo-3* promoter derived from a myosin gene expressed in body wall muscle cells and it responds to Aβ expression with paralysis (Wu and Luo, 2005). Numerous studies have shown that transgenic *C. elegans* expressing Aβ peptides can be widely used to screen candidate compounds for alleviating or treating AD.

It is well known that moderate alcohol intake has numerous benefits. Furthermore, our previous study explored the effects of long-term ethanol exposure on *C. elegans*. We found that long-term exposure to low concentrations of ethanol increased the lifespan of *C. elegans* worms via the insulin/IGF-1 signaling pathway (Yu et al., 2011). Therefore, in this study, we used the transgenic *C. elegans* CL4176 as an AD model to study the effect of ethanol on Aβ toxicity. We found that ethanol at low concentrations was effective at delaying Aβ toxicity. Further research on the mechanism of ethanol alleviating the toxicity of Aβ indicated that low concentrations of alcohol delayed the paralysis of worms to attenuate Aβ toxicity by increasing lysosomal mass and upregulating the expression of small heat shock protein genes.

## Materials and Methods

### *C. elegans* Strains and Maintenance

CL4176 (dvIs27 [*myo-3p*:A-Beta (1-42):*let-851* 3′UTR) + *rol-6* (*su1006*)] X. Rollers), TJ356 (zIs356 [*daf-16p*:*daf-16a/b*:GFP + *rol-6*(*su1006*)] were obtained from the Caenorhabditis Genetics Center (University of Minnesota, MN, United States).

Worms were cultured on nematode growth medium (NGM) agar plates and fed *Escherichia coli* OP50, according to the standard protocol (Stiernagle, 2006). To produce aged-synchronized worms, hermaphrodite worms during the egg-laying period were transferred to NGM agar plates and used to lay eggs. After 2 h, gravid adult hermaphrodite worms were removed from the plates, and their eggs were used for further experiments.

### Worm Paralysis Assay

Worms were synchronized and cultured on NGM agar plates containing different concentrations of ethanol at 16°C for 48 h. Then, the temperature was increased to 25°C, and nematodes appeared to have a paralyzed phenotype. The worms that were unable to move their body, and could only move their head when administered touch stimulation were scored as paralyzed (Li et al., 2018). The paralysis of the worms was recorded every 2 h until all worms were paralyzed. The experiment was performed three times (with more than 100 nematodes per group).

### Measurement of Fertility and Body Length of *C. elegans*

For the measurement of fecundity, the worms were cultured on NGM agar plates containing different concentrations of ethanol since birth. After reaching the reproductive period, the worms were transferred to a new NGM agar plate every 24 h until eggs were laid. The eggs on each plate were counted after they developed to the L2-L3 stages. The experiment was performed at least three times.

For the measurement of body length, the nematodes were cultured on NGM agar plates containing different concentrations of ethanol since birth. After the worms were cultured for 48 h, they were anesthetized with 50 mM sodium azide and stretched by eyebrows. The assay was performed under a microscope with measurements every 48 h until the adult stage was reached. The experiment was performed at least thrice.

### Treatment of Acetic Acid and Acetaldehyde

Worms were synchronized and cultured on NGM agar plates containing different concentrations of acetic acid or acetaldehyde (Chemical Industry Group, Beijing, China) at 16°C for 48 h. Then, the temperature was increased to 25°C, the paralysis of worms was observed every 2 h until all worms were paralyzed.

### Administration of Chloroquine

Worms were synchronized and cultured on NGM agar plates containing 1% ethanol or without ethanol at 16°C for 48 h. The worms were then transferred to NGM agar plates with 1% ethanol or without ethanol and containing 100 μM chloroquine (MedChemExpress, United States) or without chloroquine. When the temperature was increased to 25°C the paralysis of worms was observed every 2 h until all worms were paralyzed.

### Quantitative Real-Time PCR

Worms were synchronized and cultured on NGM agar plates containing 1% ethanol or without ethanol at 16°C for 48 h. After increasing the temperature to 25°C for 40 h, worms were obtained and washed with M9 buffer (42 mM Na_2_HPO_4_, 22 mM KH_2_PO_4_, 85.5 mM NaCl, 1 mM MgSO_4_), and total mRNA was extracted using Trizol reagent (TransGen Biotech, Beijing, China). The mRNA was transcribed to cDNA using TransScript One-Step gDNA Removal and cDNA Synthesis Supermix (TransGen Biotech, Beijing, China). Reverse transcripted cDNA was used as a template for quantitative PCR performed using TransStart Top Green qPCR SuperMix (TransGen Biotech, Beijing, China). The samples were analyzed using a real-time PCR instrument (ABI7500, Applied Biosystems, United States). Three independent experiments were performed (approximately 500 worms per group). Actin (*act-1*) was used as a housekeeping gene to normalize gene expression. The ΔΔCT method was used to quantify the relative levels of gene expression (Zhou et al., 2018). The gene-specific primers used were listed in Table 1.

**TABLE 1:** Lists of primers of target genes.

Gene	Forward primer	Reverse primer
*act-1*	5′-CCAGGAATTGCTGATCGTATGCAGAA-3′	5′-TGGAGAGGGAAGCGAGGATAGA-3′
*amyloid-*β	5′-CCGACATGACTCAGGATATGAAGT- 3′	5′-CACCATGAGTCCAATGATTGCA-3′
*daf-16*	5′-TTTCCGTCCCCGAACTCAA-3′	5′-ATTCGCCAACCCATGATGG-3′
*sod-3*	5′-TTCGAAAGGGAATCTAAAAGAAG-3′	5′-GCCAAGTTGGTCCAGAAGATAG-3′
*hsf-1*	5′-TTGACGACGACAAGCTTCCAGT-3′	5′-AAAGCTTGCACCAGAATCATCCC-3′
*hsp-16.1*	5′-CCACTATTTCCGTCCAGCTC-3′	5′-TGGAGAGCCTCTGCAAACTG-3′
*hsp-16.2*	5′-CTGCAGAATCTCTCCATCTGAGTC-3′	5′-AGATTCGAAGCAACTGCACC-3′
*hsp-16.49*	5′-GTCAAATCTGCAATTTCGAATG-3′	5′-CAAAATTAATGGGAATAGAACGAG-3′
*aip-1*	5′-GATTCAATCACATCAATCGCG-3′	5′-TGTTGTAGAGAACGAGCCAGAG-3′
*sip-1*	5′-CGGGTTCAGCAAGAGATCG-3′	5′-GATGGTCATCTGTCCTTCCTTG-3′
*mtl-1*	5′-GGAGGCCAGTGAGAAAAAATG-3′	5′-GCTTCTGCTCTGCACAATGAC-3′
*hsp-70*	5′-GAAGGGACGACTCTCTCAAGCT-3′	5′-CGATCTCGTTGTGCTGCG-3′
*hsp-12.6*	5′-TGGCCACTTCAAAAGGGAG-3′	5′-CTCTTTTGGGAGGAAGTTATGG-3′

### Nucleus Localization of DAF-16

Transgenic *C. elegans* strain TJ356 expressing a DAF-16:GFP reporter was synchronized and cultured on NGM agar plates containing 1% ethanol or without ethanol at 20°C for 3 days. The nematodes were anaesthetized with 50 mM sodium azide, fixed to glass slides containing 5% agar, and observed using an inverted fluorescence microscope (IX73, Olympus, Japan) with an excitation filter of 470–495 nm and an emission filter of 510–550 nm. The amount of DAF-16 in the nucleus of worms was determined using Image J software. The experiment was performed three times (with at least 15 nematodes per group).

### Thioflavin T Staining

Worms were synchronized and cultured on NGM agar plates containing 1% ethanol or without ethanol at 16°C for 48 h. The worms were then transferred to NGM agar plates with 1% ethanol or without ethanol and containing 100 μM chloroquine or without chloroquine. Following incubation at 25°C for 40 h, worms were obtained and washed three times with M9 buffer. Then, the worms were fixed with 4% paraformaldehyde/phosphate-buffered saline (pH 7.4) at 4°C for 24 h. Later, 5% β- mercaptoethanol, 1% Triton X-100, and 125 mM Tris-HCl (pH 7.5) were used to permeate the worms at 37°C for 24 h. The worms were then stained with 0.125% Thioflavin T (SECOMA, Beijing, China)/50% ethanol for 15 min, and destained with 50% ethanol for 5 min. Next, the worms were placed on a slide with phosphate-buffered saline and mounting solution (Solarbio, Beijing, China) and covered with a cover slide (Fay et al., 1998; Abbas and Wink, 2010). The fluorescence images were captured using a confocal laser microscope (LSM710, Zeiss, Germany) with an excitation filter of 488 nm. The experiment was carried out three times (at least 15 nematodes per group).

### Western Blotting

Worms were synchronized and cultured on NGM agar plates containing 1% ethanol or without ethanol at 16°C for 48 h. The worms were then transferred to NGM agar plates with 1% ethanol or without ethanol and containing 100 μM chloroquine or without chloroquine. Following incubation at 25°C for 40 h, worms were obtained and washed three times with M9 buffer. The worms were lysed by sonication in RIPA buffer (Millipore, United States) containing protease inhibitors (Solarbio, Beijing, China). The concentration of extracted protein was quantified with a Bioepitope Bicinchoninic Acid Protein Assay Kit (Bioworld, United States). Equal amounts of protein were loaded onto Tris-Tricine gels. Proteins were transferred to polyvinylidene fluoride membranes (Solarbio, Beijing, China). The primary antibodies used for immunoblotting were anti-β-Amyloid 1-16 (6E10) (BioLegend, San Diego, CA, United States) diluted 1:500 and anti-β-actin (Santa Cruz Biotechnology, Dallas, Texas, United States) diluted 1:1,000. The secondary antibodies used were horseradish peroxidase goat anti-mouse (AB Clonal, Wuhan, China) and horseradish peroxidase goat anti-rabbit (AB Clonal, Wuhan, China) diluted 1:5,000. The proteins on the polyvinylidene fluoride membrane were detected with an enhanced chemiluminescence reagent (NCM Biotech, Suzhou, China). Image J software was used to quantify band intensity.

### Staining of Lysosomes

Worms were synchronized and cultured on NGM agar plates containing 1% ethanol or without ethanol at 16°C for 48 h. Following incubation at 25°C for 40 h, worms were obtained, washed three times with M9 buffer, and immersed in M9 buffer containing 40 μM LysoTracker Red DND 99 (Solarbio, Beijing, China). Staining was carried out for 1 h at 20°C in the dark. The nematodes were then transferred onto NGM agar plates for 1 h at 20°C in the dark (Sun et al., 2020). The worms were anesthetized with 50 mM sodium azide. Fluorescence images were captured using an inverted fluorescence microscope (IX73, Olympus, Japan) with an excitation filter of 530–550 nm and an emission filter of 575–625 nm. Image J was used to analyze the fluorescence intensity of lysosomes in *C. elegans*. Three independent experiments were performed (with at least 15 worms per group).

### Proteasomal Activity

Worms were synchronized and cultured on NGM agar plates containing 1% ethanol or without ethanol at 16°C for 48 h. After increasing the incubation temperature to 25°C for 40 h, worms were obtained and washed three times with M9 buffer. Then, worms were frozen at −80°C and lysed by sonication with lysis buffer (50 mM HEPES pH 7.5, 5 mM EDTA, 150 mM NaCl, 1% Triton X-100). The concentration of extracted protein was determined using Bioepitope Bicinchoninic Acid Protein Assay Kit (Bioworld, United States). Proteasome chymotrypsin-like activity was assayed using the fluorogenic peptide Suc-LLVY-AMC (Sigma-Aldrich, Germany). The soluble protein was reacted with proteasomal activity assay buffer containing 50 mM HEPES pH 7. 5, 5 mM EDTA, 150 mM NaCl, 1% Triton X-100, 2 mM ATP, 2 mM DTT, and 25 μM Suc-LLVY-AMC at 37°C for 30 min in the dark. The fluorescence level was measured at an excitation wavelength of 340 nm and an emission wavelength of 465 nm using a microplate reader (Infinite 200 PRO, TECAN, Switzerland). The proteasome activity was calculated as the difference between total activity and residual activity with 5 μM MG132 (Aladdin, Shanghai, China), which was used to inhibit proteasome activity (Dammer et al., 2011; Vilchez et al., 2012; Shashova et al., 2014). The experiment was performed thrice (approximately 1,000 nematodes per group).

### RNAi of *daf-16*

We designed primers for *daf-16* (forward primer: 5′-ATGC GAATTCAGAATGAAGGAGC- 3′, reverse primer: 5′-TTAC AAATCAAAATGAATATGCTGC- 3′) and used the cDNA obtained by reverse transcription of the extracted RNA as a template for PCR. The PCR product of *daf-16* was cloned into the pPD129.36 vector and expressed in the competent HT115. CL4176 worms were fed *Escherichia coli* HT115 expressing pPD129.36-*daf-16* to perform *daf-16* RNAi experiment. Control nematodes were fed *Escherichia coli* HT115 containing empty pPD129.36 vector.

Worms were synchronized and cultured on NGM agar plates containing empty vector, *daf-16* RNAi bacteria or *daf-16* RNAi bacteria and ethanol at 16°C for 48 h. When the temperature was increased to 25°C, the paralysis of worms was observed every 2 h until all worms were paralyzed.

### Statistical Analysis

Statistical analysis was performed using GraphPad Prism software (GraphPad, La Jolla, CA, United States). The log-rank (Mantel-Cox) test was used to analyze paralysis. The comparison of the treatment and control groups in other experiments was performed using the *t*-test. The difference in statistical data is shown with *p*-value; *p* < 0.05 (^∗^), *p* < 0.01 (^∗∗^), and *p* < 0.001 (^∗∗∗^) were regarded as significant.

## Results

### Ethanol at Low Concentration Delays the Paralysis of *C. elegans*

**FIGURE 1:**
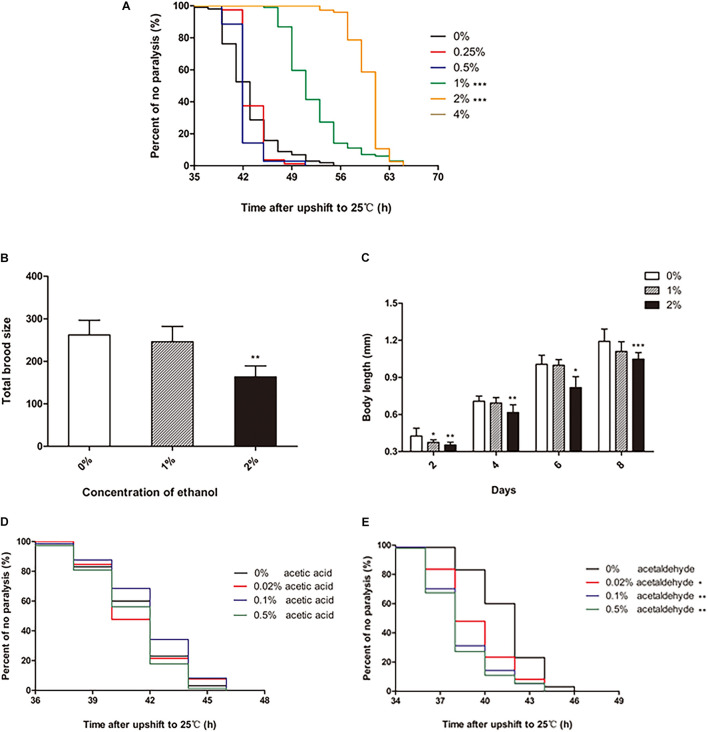
Effect of ethanol on the paralysis of CL4176 *C. elegans*. **(A)** Worms were treated with different concentrations of ethanol [0.25%, 0.5%, 1%, 2%, 4%, 6%, 8%, 10% (v/v)] or 0% as control. Data represents the paralysis analysis of *C. elegans* at each concentration of ethanol, *n* > 100. **(B)** Total brood size was measured by counting the number of eggs that each worm laid. Each column represents mean ± SD, *n* > 10. **(C)** Body length was determined by microscopy on the days of the lifespan as shown, *n* > 10. **(D)** Worms were treated with different concentrations of acetic acid [0.02, 0.1, and 0.5% (v/v)] or 0% as control. Data represents the paralysis analysis of *C. elegans* at each concentration of acetic acid, *n* > 100. **(E)** Worms were treated with different concentrations of acetaldehyde [0.02, 0.1, and 0.5% (v/v)] or 0% as control. Data represents the paralysis analysis of *C. elegans* at each concentration of acetaldehyde, *n* > 100. *p < 0.05, **p < 0.01 and ***p < 0.001.

To test the effect of ethanol on the paralysis of *C. elegans*, different concentrations of ethanol [0.25, 0.5, 1, 2, 4, 6, 8, and 10% (v/v)] were administered to the worms on NGM, starting from the egg stage. Ethanol concentrations of 0.25 and 0.5% did not have a significant effect on the paralysis of *C. elegans* compared with that in the control (not containing ethanol). However, 1 and 2% ethanol significantly delayed paralysis in *C. elegans* (Figure 1A). 1% ethanol prolonged the average paralysis time by 9.29 h, and 2% ethanol delayed the mean paralysis time by 17.06 h. The worms appeared to develop incompletely when ethanol concentration increased from 4 to 10%.

The data showed that certain concentrations of ethanol influenced the development of *C. elegans*. Therefore, we explored the effect of ethanol on nematode development. Fertility and body length are the two main factors that determine worm development. Compared to the control group, the worms treated with 1% ethanol did not show a difference in total brood size. However, for worms treated with 2% ethanol, the number of eggs laid was significantly reduced compared to that in the control group (Figure 1B). Regarding body length, the worms treated with 2% ethanol were shorter than those in the control group at 2, 4, 6, and 8 days (Figure 1C). Analysis of fertility and body length showed that 2% ethanol delayed the development of worms. Therefore, we treated the worms with 1% ethanol for subsequent experiments.

We further explored whether the effect of ethanol on delaying nematode paralysis is due to ethanol itself or its metabolites acetic acid or acetaldehyde. So we tested the effect of different concentrations of acetic acid and acetaldehyde on the paralysis of the worms. The mean paralysis time of the worms was not different between the acetic acid treatment group [0.02, 0.1, and 0.5% (v/v)] and the control group (Figure 1D). However, 0.02, 0.1, and 0.5% acetaldehyde significantly decreased the average paralysis time of nematodes compared to the control group (Figure 1E).

### Aβ Oligomers Are Reduced in Transgenic *C. elegans* Treated With Ethanol

**FIGURE 2:**
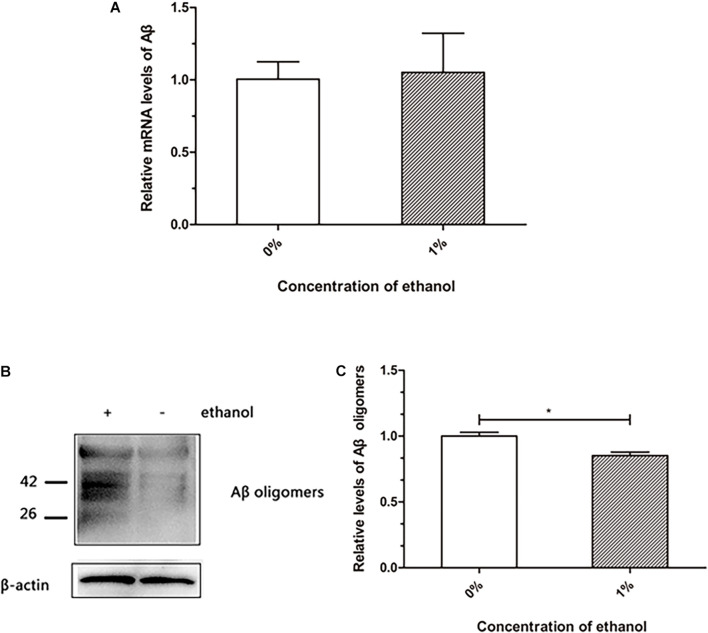
Effect of ethanol on Aβ production and aggregation of *C. elegans*. **(A)** Relative *A*β mRNA level was determined by using quantitative real-time PCR, *n* = 500. **(B)** Aβ oligomers were determined by western blot, *n* = 1,000. **(C)** The relative level of Aβ oligomer expression was quantified by Image J software. The bar graph represents mean ± SD. *p < 0.05.

The transgenic nematode strain CL4176 is able to express human Aβ, which accumulates to form deposits in the body wall muscle cells, resulting in worm paralysis. We investigated whether ethanol affected Aβ production and accumulation in *C. elegans*. The qPCR analysis of *A*β mRNA did not reveal any difference between the control and ethanol-treated worms (Figure 2A). Western blotting results suggested that Aβ oligomers were reduced in the worms treated with 1% ethanol compared to those in the control group (Figure 2C).

### Lysosomes Are Increased in Nematodes Treated With Ethanol

**FIGURE 3:**
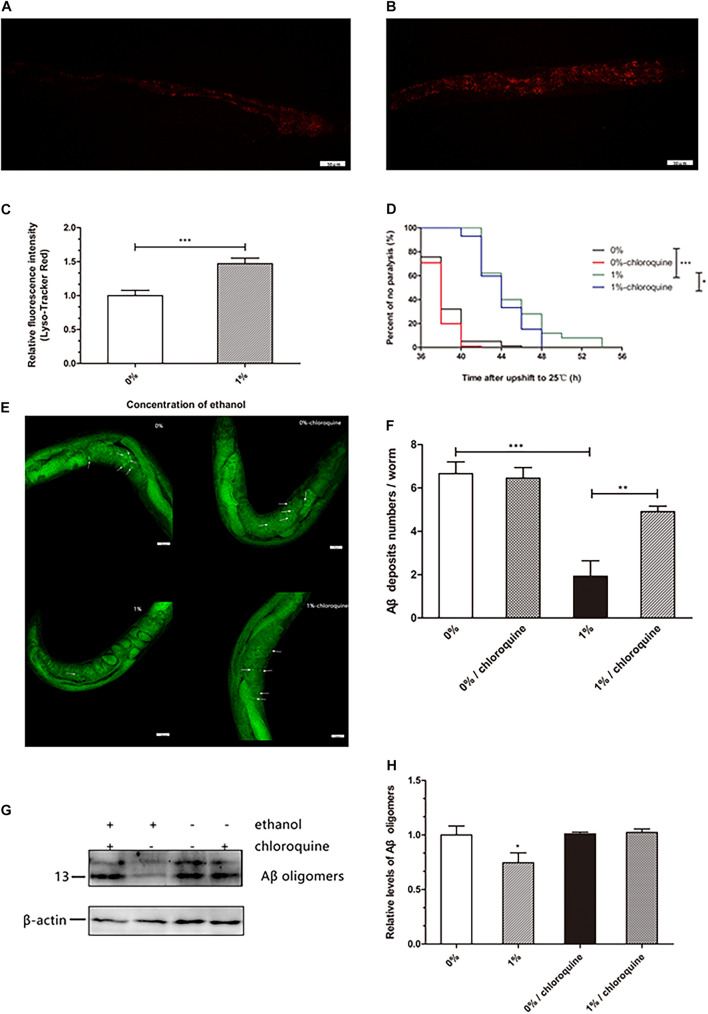
Effect of ethanol on lysosome mass of *C. elegans*. The measurement of lysosome mass was performed by LysoTracker Red staining. The photo was taken under 20 × objective with a fluorescent microscope. The number of lysosomes is shown by the red fluorescence in the control group **(A)** and 1% ethanol group, *n* = 15 **(B)**. **(C)** Image J was used to determine the fluorescence intensity of lysosomes. The bar graph shows mean ± SD. **(D)** Worms were cultured on 1% ethanol or without ethanol NGM agar plates containing 100 μM chloroquine or without chloroquine. Data represents the paralysis analysis of *C. elegans* treated with chloroquine, *n* > 100. **(E)** Aβ deposits were measured by Thioflavin T staining, the photo was taken using a 20 × objective with confocal microscopy, *n* = 15. The arrowheads point out the Aβ deposits. **(F)** The number of beta amyloid protein deposits was calculated by mean ± SD. **(G)** Aβ oligomers were determined by western blot after worms were fed chloroquine, *n* = 1,000. **(H)** The relative level of Aβ oligomer expression was quantified by Image J software. The bar graph represents mean ± SD. *p < 0.05, **p < 0.01 and ***p < 0.001.

Lysosomes are important organelles that clear misfolded and damaged proteins to maintain an organism’s proteostasis. We examined whether ethanol increased lysosomal mass to clear Aβ in worms. The results of a lysosome quantification assay performed using LysoTracker Red, a fluorescent probe that specifically binds lysosomes and other acidic compartments, suggested that the number of lysosomes in *C. elegans* treated with ethanol was increased compared to that in control worms (Figures 3A–C).

Worms treated with ethanol were cultured on NGM agar plates in the presence of 100 μM chloroquine to inhibit lysosome acidification. We found that 1% ethanol delayed the mean paralysis time of worms by 17.7% compared to that in the control group. However, in worms exposed to 100 μM chloroquine and 1% ethanol, the average paralysis time was significantly shorten compared to that in the 1% ethanol treatment group (Figure 3D). These results showed that inhibition of lysosome acidification partly attenuated the effect of ethanol on the paralysis of nematodes.

Thioflavin T (a dye that especially binds to beta-amyloid) staining assay showed that ethanol treatment significantly reduced the number of Aβ aggregates (1.93 ± 0.71) compared to that in the control group (6.66 ± 0.54). However, after the nematodes were treated with chloroquine, ethanol was no longer able to reduce the number of Aβ aggregates (4.91 ± 0.26) (Figure 3F). Western blotting also showed similar results. After the nematodes were treated with chloroquine, ethanol was no longer able to reduce Aβ oligomers (Figure 3G).

### Effect of Ethanol on Proteasome Activity in *C. elegans*

**FIGURE 4:**
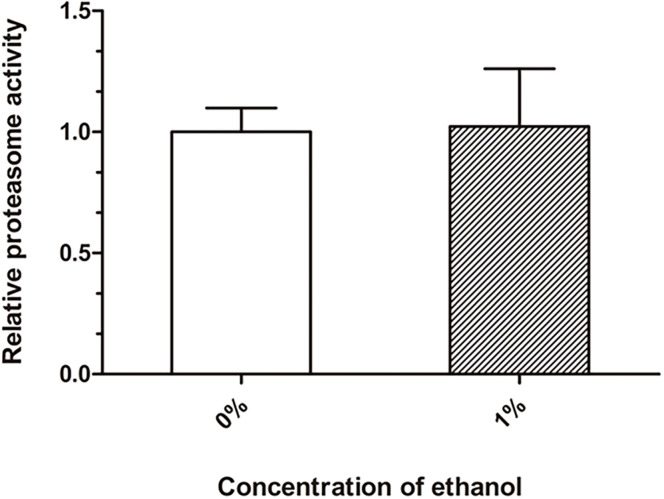
Effect of ethanol on proteasome activity of *C. elegans*. The chymotrypsin-like proteasome activity was determined by the Suc-LLVY-AMC fluorescent substrate. Graphs represent mean ± SD, *n* = 1,000.

Proteasome degradation is the main degradation pathway for the removal of abnormal cellular proteins, including misfolded and damaged proteins. We performed a chymotrypsin-like proteasome activity assay to quantify the proteasome function. There was no significant difference in the proteasome activity between the ethanol-treated group and control group (Figure 4).

### The Effect of DAF-16 on Ethanol-Regulated Amyloid-Beta Toxicity Is Crucial in *C. elegans*

**FIGURE 5:**
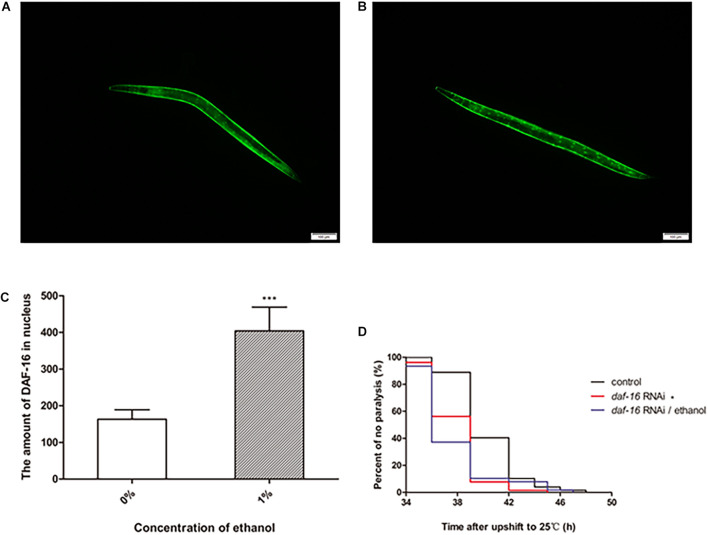
The effect of DAF-16 on ethanol-regulated amyloid-beta toxicity of *C. elegans*. Fluorescent microscopy was used to measure DAF-16 localization in TJ356 transgenic worms treated without ethanol **(A)** and with ethanol, *n* = 15 **(B)**. The photo was taken using a 10 × objective with a fluorescent microscope. **(C)** The amount of nuclear DAF-16::GFP was evaluated by Image J between ethanol-treated group and the control. The graph represents the mean ± SD. **(D)** Worms were fed with *daf-16* RNAi bacteria or *daf-16* RNAi bacteria and ethanol or empty vector bacteria as control, *n* > 100. Data represents the paralysis analysis of *C. elegans* at each condition. *p < 0.05 and ***p < 0.001.

DAF-16 is an important transcription factor involved in regulating several biological processes in *C. elegans*. DAF-16 is distributed in the cytoplasm under normal physiological conditions. When activated, DAF-16 relocates to the nucleus. To determine whether ethanol activated DAF-16 translocation, we used the TJ356 strain to measure DAF-16 nuclear localization. The results showed that treatment with 1% ethanol significantly induced DAF-16 nuclear localization (Figures 5A–C).

We also determined the role of *daf-16* in nematode paralysis by RNA interference. And the result showed that *daf-16* RNAi significantly shortened the average paralysis time of nematodes compared with the control group. After *daf-16* RNAi, ethanol no longer had the effect of prolonging the mean paralysis time of the worms (Figure 5D).

### Ethanol Affects Gene Expression Involving the Stress Response in *C. elegans*

**FIGURE 6:**
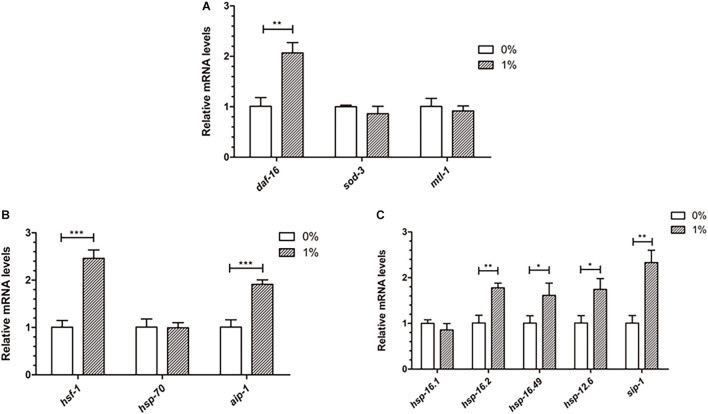
Effect of ethanol on the expression of stress resistance genes in worms. **(A)** The expression of *daf-16* and downstream target genes (*sod-3*, *mtl-1*) was performed by qPCR, *n* = 500. **(B)** The relative mRNA level of *hsf-1* and downstream target genes (*hsp-70*, *aip-1*) was measured by qPCR. **(C)** The expression of small heat shock protein genes (*hsp-16.1*, *hsp-16.2*, *hsp-16.49*, *hsp-12.6*, *sip-1*), common downstream target genes, was determined by using qPCR. Graphs represent mean ± SD. *p < 0.05, **p < 0.01 and ***p < 0.001.

Ethanol, a chemical stressor, may activate genes related to stress to prevent Aβ toxicity in *C. elegans*. DAF-16 and HSF-1 are two important transcription factors that induce stress resistance in nematodes. We measured the mRNA levels of *daf-16*, *hsf-1* and their down-stream genes to determine whether ethanol delayed paralysis through inducing these stress response pathways. The qPCR analysis of these genes showed that *daf-16* and *hsf-1* were upregulated in worms treated with ethanol (Figures 6A,B). Moreover, the downstream target genes (*aip-1*, *sip-1*, *hsp-16.2*, *hsp-16.49*, *hsp-12.6*) of *daf-16* and *hsf-1* were significantly upregulated in *C. elegans* treated with ethanol (Figures 6B,C). The mRNA levels of *sod-3*, *mtl-1*, *hsp-70* and *hsp-16.1* did not differ between ethanol-treated worms and control worms (Figures 6A–C).

## Discussion

It is well-known that alcohol has numerous beneficial and harmful effects. Some studies have suggested that high alcohol concentrations cause neurotoxicity and cognitive and sleep impairments, as well as mood disorders, such as depressive-like behaviors (Tizabi et al., 2018). Others have shown that low-moderate alcohol use is associated with a decreased risk of dementia or cognitive decline (Peters et al., 2008). Meanwhile, we found that long-term exposure to low concentrations of ethanol could prolong the lifespan of *C. elegans* in an insulin/IGF-1 pathway-dependent manner in a previous study. In this study, we examined the effect of ethanol on the paralysis of the transgenic *C. elegans* strain CL4176 expressing Alzheimer’s Aβ1-42. Our data revealed that ethanol significantly delayed paralysis at low concentrations of 1 and 2% in CL4176 (Figure 1A), while the total brood size and body length were obviously reduced in the worms treated with 2% ethanol (Figures 1B,C). The results demonstrated that 2% ethanol delayed the development of *C. elegans*. Therefore, we used 1% ethanol as the optimal concentration. Acetic acid and acetaldehyde are two of the main metabolites of ethanol. We also measured the effect of these ethanol metabolites on nematode paralysis to determine whether ethanol delayed nematode paralysis by itself or due to its breakdown to these metabolites. Acetic acid at concentrations of 0.02, 0.1, and 0.5% did not delay the paralysis of worms compared to the control group (Figure 1D). However, 0.02, 0.1, and 0.5% acetaldehyde significantly reduced the mean paralysis time of nematodes vs. the control (Figure 1E). Therefore, the results suggested that the effect of ethanol in delaying nematode paralysis is due to ethanol itself rather than its metabolites.

Senile plaques consist of Aβ, which is one of the hallmarks in the brain of AD patients. Aβ is hydrolyzed from amyloid precursor protein into a polypeptide of 39–43 residues, which is toxic and easily aggregates (Iwatsubo et al., 1994). Therefore, understanding the production and aggregation of Aβ is vital for the prevention and treatment of AD. Our study demonstrated that ethanol did not reduce *A*β production at the mRNA level in *C. elegans* (Figure 2A). Western blot assays showed that Aβ oligomers were reduced in nematodes treated with ethanol (Figure 2B). These results indicated that ethanol reduced Aβ aggregation to delay worm paralysis.

Accumulation of misfolded and damaged proteins may trigger diseases related to proteostasis, such as Alzheimer’s, Parkinson’s, and Huntington’s disease (Regitz et al., 2016). The effective degradation of abnormal proteins is performed to maintain proteostasis in an organism. This process is accomplished through the autophagy-lysosome or proteasome pathways (Vilchez et al., 2014; Zhang et al., 2017). Therefore, we determined the lysosome and proteasome activities to investigate whether ethanol plays a role in activating protein degradation in *C. elegans* CL4176. LysoTracker Red, a red fluorescent probe, is used for staining the lysosomes and other acidic compartments of live cells. The results of staining assays indicated that the number of lysosomes was increased in *C. elegans* treated with ethanol compared to the control group (Figure 3A). Chloroquine, a lysosome inhibitor, was administered to worms to inhibit lysosomal acidification. The average paralysis time of nematodes treated with ethanol and chloroquine was shorter than that of nematodes treated with only ethanol (Figure 3D). Thioflavin T, an amyloid-specific fluorescent dye, can detect Aβ deposits in worms. Thioflavin T staining suggested that ethanol significantly reduced Aβ deposits in *C. elegans*. However, when given chloroquine, Aβ deposits of worms could not be reduced with ethanol treatment (Figure 3E). After feeding the nematodes with chloroquine, the amount of Aβ oligomers determined by Western blotting showed similar results (Figure 3H). These results suggested that inhibition of lysosome acidification could decrease the effect of ethanol on nematode paralysis and Aβ protein degradation. In addition, one study has demonstrated that acute treatment of neuronal cells with ethanol reduces ASIC1a protein expression via an autophagy-lysosome-dependent pathway (Zhou et al., 2019). However, the measurement of chymotrypsin-like proteasome activity using Suc-LLVY-AMC, a fluorogenic substrate of the proteasome, did not show a difference between the nematodes treated with ethanol and control worms (Figure 4). These results demonstrated that ethanol might increase lysosomal biogenesis to promote Aβ degradation, thereby delaying paralysis in *C. elegans*.

Ethanol, regarded as an exogenetic stressor, may stimulate organisms to respond to the environment. DAF-16 and HSF-1 are two important transcription factors related to stress, metabolism and longevity in *C. elegans* (Hesp et al., 2015; Brunquell et al., 2016). Enhancement of their activity leads to induction of the expression of downstream genes to resist harmful conditions. We used transgenic TJ356 worms, that express a fused GFP reporter protein with DAF-16, to observe the DAF-16 distribution in nematodes. The results showed that ethanol induced DAF-16 nuclear translocation in TJ356 worms (Figures 5A,B). RNAi of *daf-16* also showed that *daf-16* played an important role in delaying the paralysis of the worms. And the effect of ethanol in delaying nematode paralysis is dependent on DAF-16 (Figure 5D).

The qPCR analysis of genes involved in stress resistance indicated that ethanol increased the expression of *daf-16* and *hsf-1* (Figures 6A,B). Moreover, *aip-1*, a downstream target gene of *hsf-1*, was upregulated in worm-treated with ethanol (Figure 6B). The expression of small heat shock protein genes, including *hsp-16.2*, *hsp-16.49*, *hsp-12.6*, and *sip-1*, common downstream target genes of *daf-16* and *hsf-1*, were increased significantly in ethanol- treated *C. elegans* (Figure 6C). Some studies found that DAF-16 and HSF-1 promote the expression of small heat shock protein genes, including *hsp-16.1*, *hsp-16.49*, *hsp-12.6*, and *sip-1*. Small heat shock proteins bind to unfolded proteins and prevent their aggregation (Ao-Lin Hsu and Murphy, 2003; Hesp et al., 2015). These results showed that ethanol may play an important role in reducing Aβ-induced toxicity through HSF-1- and DAF-16-dependent pathways.

The present study indicates that ethanol delays paralysis and reduces Aβ oligomers in *C. elegans*. In addition, ethanol increases lysosome mass, induces DAF-16 nuclear translocation, and upregulates the expression of *daf-16*, *hsf-1*, and small heat shock protein genes in the worms. Based on these results, ethanol plays a crucial role in preventing Aβ toxicity in this transgenic *C. elegans* model strains of AD. The mechanism of action of low concentrations of ethanol in promotion of Aβ degradation and reduction of Aβ aggregation appears to be through increasing levels of lysosomes and through DAF-16- and HSF-1-dependent pathways.

## Data Availability Statement

The original contributions presented in the study are included in the article/supplementary material, further inquiries can be directed to the corresponding author/s.

## Author Contributions

JM and SB designed the study. WW, ZZ, ML, and ZC performed the experiments. JW, YZ, LA, and YW collected the data. JM, SB, and SX analyzed the experiments. SB wrote the manuscript. JM and XF edited the manuscript. All authors contributed to the article and approved the submitted version.

## Conflict of Interest

The authors declare that the research was conducted in the absence of any commercial or financial relationships that could be construed as a potential conflict of interest.

## Publisher’s Note

All claims expressed in this article are solely those of the authors and do not necessarily represent those of their affiliated organizations, or those of the publisher, the editors and the reviewers. Any product that may be evaluated in this article, or claim that may be made by its manufacturer, is not guaranteed or endorsed by the publisher.
